# Ya‐Jie‐Sha‐Ba Decoction Regulates Arachidonic Acid Metabolism to Treat Alcohol‐Associated Liver Disease by Activating PPARα–CYP4A14 Axis: Insights From Multi‐Omics Analysis and Experimental Validations

**DOI:** 10.1111/jcmm.71385

**Published:** 2026-09-29

**Authors:** Dehong Ma, Xia Chen, Huixian Dao, Hongyu Deng, Yingcheng Guo, Hui Yang, Yuanyuan Chen, Congmei Zhang, Yuan Long, Weibo Wen, Yinghong Zhao, Huantian Cui

**Affiliations:** ^1^ First School of Clinical Medicine Yunnan University of Chinese Medicine Kunming China; ^2^ Xishuangbanna Dai Autonomous Prefecture Dai Medicine Hospital Jinghong China; ^3^ Department of Spleen and Stomach Diseases Yunnan Provincial Hospital of Traditional Chinese Medicine Kunming China

**Keywords:** alcohol‐associated liver disease, arachidonic acid metabolism, methyl palmitate, oxidative stress, PPARα–CYP4A14 axis, Ya‐Jie‐sha‐Ba decoction

## Abstract

Ya‐Jie‐Sha‐Ba decoction (YJSB), a traditional Dai medicinal formulation, has been widely used by the Dai ethnic group and has shown efficacy in alleviating alcohol‐associated liver disease (ALD). However, the molecular mechanism of YJSB in the management of ALD remains unclear. Building on evidence that YJSB ameliorates ALD, we combined transcriptomics and untargeted metabolomics to elucidate the molecular mechanisms responsible for its anti‐ALD effects. We established an ALD mouse model and treated animals with YJSB to evaluate its therapeutic effects. We then performed integrated transcriptomic and untargeted metabolomic analyses to identify pathways and molecules associated with YJSB treatment. Using UPLC‐Q‐TOF‐MS, we identified YJSB prototype components present in plasma. We screened the major plasma‐detected constituent for hepatoprotective activity in an in vitro model of alcohol‐induced hepatocyte injury. Finally, we validated the molecular target of this active component using molecular docking, cellular thermal shift assay (CETSA), drug affinity responsive target stability (DARTS), and pharmacological inhibition assays. The results showed that YJSB markedly reduced hepatic injury and lipid accumulation in ALD mice and simultaneously decreased oxidative stress and inflammation. Integrated multi‐omics analysis identified arachidonic acid metabolism as a potential pathway mediating YJSB's protective effects. In particular, YJSB robustly increased the expression of CYP4A14, a key enzyme in this pathway. We identified methyl palmitate as the major active component of YJSB that mitigated alcohol‐induced hepatocyte injury in vitro. Methyl palmitate decreased oxidative stress and enhanced CYP4A14 expression in injured hepatocytes. Molecular docking demonstrated a preferable binding affinity between methyl palmitate and PPARα. CETSA and DARTS further validated this interaction by showing that methyl palmitate increased the thermal stability and proteolytic resistance of PPARα. Importantly, pharmacological inhibition of PPARα completely abolished the hepatoprotective effects of methyl palmitate in vitro. In conclusion, YJSB effectively reduces liver injury in ALD mice. Mechanistically, its active component methyl palmitate activates PPARα, thereby upregulating CYP4A14, reshaping arachidonic acid metabolism, and attenuating alcohol‐induced oxidative stress in hepatocytes.

**Abbreviations**
AA: arachidonic acid
ADH: alcohol dehydrogenase
ALD: alcohol‐associated liver disease
ALDH: aldehyde dehydrogenase
ALT: alanine aminotransferase
AST: aspartate aminotransferase
CETSA: cellular thermal shift assay
COX: cyclooxygenase
CYP: cytochrome P450
CYP2E1: cytochrome P450 2E1
DARTS: drug affinity responsive target stability
DEGs: differentially expressed genes
EETs: epoxyeicosatrienoic acids
FC: fold change
H&E: haematoxylin and eosin
KEGG: Kyoto Encyclopedia of Genes and Genomes
LOX: lipoxygenase
MASLD: metabolic dysfunction‐associated steatotic liver disease
MCD: methionine‐choline‐deficient
MP: methyl palmitate
NC: normal control
PC: positive control
PCA: principal component analysis
PLS‐DA: partial least squares discriminant analysis
ROS: reactive oxygen species
TC: total cholesterol
TCM: Traditional Chinese Medicine
TG: triglyceride
TIC: total ion chromatogram
YJSB: Ya‐Jie‐Sha‐Ba decoction
YJSB‐H: YJSB high‐dose
YJSB‐L: YJSB low‐dose
YJSB‐M: YJSB medium‐dose

## 1 Introduction

Alcohol‐associated liver disease (ALD) is a common chronic liver disease arising from prolonged, excessive alcohol consumption. ALD usually begins with hepatic steatosis and may progress to various other conditions, including liver fibrosis, alcoholic hepatitis, or cirrhosis [1]. In severe cases of alcohol abuse, extensive hepatocyte necrosis can develop and lead to liver failure, posing a major threat to public health [2]. Recent epidemiological data show a growing prevalence of ALD in younger populations, with incidence rates increasing annually. Alarmingly, the 5‐year mortality rate among patients with ALD exceeds 50%, creating a substantial societal and healthcare burden [3]. Alcohol abstinence and exercise remain the most effective strategies to reduce liver injury in individuals with ALD [4]. However, it is difficult to achieve in clinical practice due to low abstinence rates and high relapse rates [5, 6]. For patients with advanced ALD including severe alcoholic hepatitis or cirrhosis, liver transplantation offers the most effective treatment, yet the number of patients awaiting transplantation continues to rise sharply each year [7]. These trends underscore the need for early prevention and timely intervention. Several agents including glucocorticoids, S‐adenosylmethionine, metadoxine, polyenylphosphatidylcholine, glycyrrhizin preparations, silymarin, reduced glutathione and bicyclol are currently used to improve liver function in early‐stage ALD. However, long‐term treatment is often limited by adverse effects that weaken patient adherence [8]. Thus, there is an urgent need to develop novel therapeutic strategies that are safe, effective, and suitable for long‐term use to improve ALD prevention and management.

Oxidative stress is widely recognized as a central pathogenic driver of ALD [9]. Under physiological conditions, hepatocytes metabolize ethanol primarily through alcohol dehydrogenase (ADH), producing acetaldehyde, which is further converted by aldehyde dehydrogenase (ALDH) into acetate and ultimately oxidized to carbon dioxide and water in extrahepatic tissues. Chronic and excessive alcohol intake disrupts this metabolic balance by inducing cytochrome P450 2E1 (CYP2E1) and impairing ALDH activity, resulting in hepatic acetaldehyde accumulation [10]. Elevated acetaldehyde depletes glutathione and increases reactive oxygen species (ROS) production, leading to lipid peroxidation and the formation of toxic acetaldehyde–lipid adducts. These adducts cause cytotoxicity and activate immune responses, thereby amplifying inflammation and fibrogenesis in ALD [11, 12]. Consistent with these mechanisms, patients with ALD commonly exhibit an imbalance between pro‐oxidant and antioxidant systems [2]. Notably, randomized controlled clinical trials report that antioxidant supplementation improves survival in individuals with severe acute alcoholic hepatitis [13]. Antioxidants such as digoxin and N‐acetylcysteine have shown therapeutic benefits across multiple stages of clinical investigation in ALD [14]. Thus, strategies that enhance hepatic antioxidant capacity offer a promising approach for ALD treatment.

Traditional Chinese Medicine (TCM) provides distinctive advantages in ALD prevention and therapy, particularly in limiting oxidative stress [15]. Several formulations including Qi‐Wei‐Jing‐Gan‐Ling [16], Ge‐Zhi‐Jie‐Jiu‐Tang [17] and Xie‐Zhuo‐Tiao‐Zhi‐Tang [18] have demonstrated efficacy against ALD via antioxidant mechanisms. Moreover, active compounds such as hydroxysafflor yellow A [19], astragaloside IV [20] and ursolic acid [21] exert hepatoprotective effects by improving lipid metabolism, reducing oxidative stress, and suppressing inflammatory responses. Continued investigation of TCM‐based therapeutics and their mechanistic basis holds significant potential for developing safe, effective, and multi‐targeted strategies for ALD management.

Ya‐Jie‐Sha‐Ba decoction (YJSB) is a distinctive Dai medicinal formulation traditionally used to protect liver and kidney function and to counteract damage caused by harmful substances, including alcohol [22]. YJSB's major constituents include eight herbs: *Dregea sinensis* Hemsl, *Arundina graminifolia* (D. Don) Hochr, *Fibraurea recisa* Pierre, *Pueraria montana var. lobata* (Willd.) Maesen & S.M.Almeida ex Sanjappa & Predeep, *Mappianthus iodoides* Hand.‐Mazz, *Anodendron nervosum* Kerr, *Clerodendrum chinense* (Osbeck) Mabb, and *Glycyrrhiza uralensis* Fisch. ex DC. The major constituents have been checked against http://www.theplantlist.org. Although recent studies report that YJSB alleviates liver injury in animal models induced by acute alcohol exposure or carbon tetrachloride [23, 24], its molecular mechanisms in ALD remain largely undefined. In this study, we established an ALD mouse model and administered YJSB to comprehensively evaluate its therapeutic effects. We then integrated transcriptomic and untargeted metabolomic analyses to identify and validate the molecular pathways through which YJSB exerts its anti‐ALD activity in vivo. In parallel, we characterized the chemical composition of YJSB and identified its prototype compounds detectable in plasma. These compounds were further evaluated in an in vitro model to confirm the molecular basis of YJSB's hepatoprotective effects. Together, our findings systematically elucidate the therapeutic actions of YJSB in ALD, identify its key bioactive constituents, and define the underlying molecular mechanisms–providing a strong theoretical and chemical foundation for its clinical application.

## 2 Methods

### 2.1 In Vivo Experiments

#### 2.1.1 Animals and Reagents

Male C57BL/6 mice (SPF grade, 6–8 weeks old, weighing 18–22 g) were obtained from Beijing Huafukang Biotechnology Co. Ltd. (production licence number: SCXK (Jing)2024–0003). All animals were housed in the Animal Experiment Center of Yunnan University of Chinese Medicine and acclimated for 1 week before experimentation. All procedures complied with national guidelines for the ethical use of laboratory animals and were approved by the Yunnan University of Chinese Medicine Ethics Committee (Approval No.: YNUCM‐XMSB‐G‐20250274).

Methyl palmitate (molecular formula: C_17_H_34_O_2_; Purity: 97%) was purchased from Shanghai Yuanye Bio‐Technology Co. Ltd. A complete list of additional reagents used in this study is provided in the Supporting Informations.

#### 2.1.2 Chemical Profiling of YJSB

YJSB was obtained from the Dai Hospital of Xishuangbanna Dai Autonomous Prefecture. One gram of YJSB was weighed and extracted with 10 mL of methanol by ultrasonication for 30 min. The mixture was then centrifuged at 3000 rpm for 10 min. The supernatant was filtered through a 0.22 μm microporous membrane, and the filtrate was transferred into an HPLC vial for analysis.

Mice were administered YJSB by intragastric gavage at a high dose for three consecutive days. Plasma was collected at 1 and 2 h after the last administration and mixed uniformly. Methanol was added to the plasma at a volume ratio of 1:3 (plasma: methanol), vortexed for 30 s, and allowed to stand at 4°C for 20 min. The mixture was then centrifuged at 12,000 × g for 15 min, and the supernatant was collected and vacuum‐dried. The residue was reconstituted in 100 μL methanol, centrifuged at 12,000 × g for 10 min, and the supernatant was collected. This supernatant was again vacuum‐dried, reconstituted in 100 μL methanol, and centrifuged at 12,000 × g for 10 min. The final supernatant was transferred to an autosampler vial for subsequent instrumental analysis. Detailed analytical methods are described in the Supporting Informations.

#### 2.1.3 Experimental Design

Sixty male C57BL/6 mice were randomly assigned to six groups (*n* = 10 per group): Normal control (NC), ALD model (ALD), Positive control (PC; 60 mg/kg silybin), and YJSB low‐, medium‐, and high‐dose groups (YJSB‐L, −M, −H; 13.6, 27.2, 54.4 g/kg). Except for the NC group, all mice underwent the Gao‐binge ALD protocol as previously described [25]. From Day 0 to Day 5, mice in the NC group received a control liquid diet, whereas mice in all other groups were given an ethanol‐containing liquid diet with ethanol concentration gradually increasing from 0% to 4% (v/v). From Day 6 to Day 19, mice continued on either the control diet or a 5% (v/v) ethanol liquid diet. We recorded body weight every 3 days. Fresh liquid diet was supplied daily and adjusted according to the previous day's intake. All animals had ad libitum access to food and water. On Day 19, mice were gavaged with either a maltodextrin solution (20 mL/kg, 45% w/v) or an ethanol solution (20 mL/kg, 31.5% v/v). Nine hours later, mice were anaesthetised with isoflurane, and blood and liver tissues were collected for downstream analyses.

#### 2.1.4 Histopathological Analysis

We fixed liver tissues in 4% paraformaldehyde, embedded them in paraffin, and sectioned and stained them with haematoxylin and eosin (H&E) to evaluate hepatic injury under light microscopy [26]. Hepatic steatosis was scored to evaluate the severity of hepatic steatosis, with the specific methodology referenced from previous studies [27, 28]. Frozen liver sections were stained with Oil Red O to assess hepatic lipid accumulation.

#### 2.1.5 Transcriptomics and Untargeted Metabolomics

We collected liver tissues from each group for transcriptomic and metabolomic analyses. Total RNA was isolated using a commercial extraction kit and submitted to Novogene Co. Ltd. for high‐throughput sequencing on the Illumina HiSeq platform. Sequencing reads were aligned with HISAT2 v2.0.5. We identified differentially expressed genes (DEGs) using DESeq2 v1.20.0 with thresholds of |log_2_ FC| > 1 and *p* < 0.05. DEGs were subsequently analysed for Kyoto Encyclopedia of Genes and Genomes (KEGG) pathway enrichment.

For untargeted metabolomics, liver tissues were homogenized in extraction buffer, sonicated, and centrifuged. Supernatants were dried under vacuum and reconstituted in 50% acetonitrile containing 2‐chloro‐L‐phenylalanine as an internal standard. Following filtration, samples were analysed by LC–MS at Novogene Co. Ltd. using chromatographic and MS conditions established in our previous studies [29]. Raw data were processed with XCMS for peak detection, filtering, and alignment. Metabolites were identified by comparison with the NovoMetDB‐UM‐V3.0 database. Data were normalized and analysed using metaX software to conduct principal component analysis (PCA) and partial least squares discriminant analysis (PLS‐DA). Differentially abundant metabolites were defined by VIP > 1, *p* < 0.05, and fold change (FC) > 1.5 or < 0.67. Identified metabolites were then subjected to KEGG pathway enrichment analysis.

### 2.2 In Vitro Experiments

#### 2.2.1 Cell Culture and Treatment

We obtained HepG2 cells from Shanghai Fuheng Biotechnology Co. Ltd., and maintained them in DMEM supplemented with 10% fetal bovine serum and 1% penicillin–streptomycin in a humidified 5% CO₂ incubator at 37°C. We seeded log‐phase cells into 96‐well plates at 1 × 10^4^ cells/well. To determine optimal treatment doses, cells were exposed to graded concentrations of ethanol (50, 100, 200, 400, and 800 mM) or methyl palmitate (3.125, 6.25, 12.5, 25 and 50 μM) for 24 h. For subsequent experiments, cells were injured with 800 mM ethanol for 24 h and then treated with 50 μM of methyl palmitate to assess its protective effects against alcohol‐induced hepatotoxicity.

#### 2.2.2 MTT Assay

Following treatment, we added 20 μL of MTT solution (5 mg/mL) to each well and incubated it for 4 h. We then removed the medium and dissolved formazan crystals in DMSO (100 μL). OD_490_ was measured using a microplate reader, and relative cell viability was calculated accordingly.

#### 2.2.3 ROS Detection

After removing the culture medium, cells were washed three times with PBS. Intracellular ROS levels were measured using the DCFH‐DA fluorescent probe. We incubated cells with 10 μM DCFH‐DA for 0.5 h (dark, 37°C), and then performed PBS wash 3 times. Cells were imaged immediately under an inverted fluorescence microscope.

#### 2.2.4 PPARα Transcriptional Activity Assay

We assessed PPARα transcriptional activity using a luciferase reporter system as previously described [30]. HepG2 cells were co‐transfected with pcDNA3.1‐PPARα (450 ng), a PPRE×3‐TK‐LUC reporter plasmid (150 ng), and a pRSV‐gal β‐galactosidase control plasmid (50 ng) using Lipofectamine 3000. After drug treatment, luciferase activity was measured and normalized to β‐galactosidase activity to determine relative PPARα transcriptional activation.

#### 2.2.5 Molecular Docking, CETSA and DARTS

We performed molecular docking between the 2D structure of methyl palmitate and the 3D crystal structure of PPARα using the CB‐DOCK2 online platform. Docking scores < −5.0 kcal/mol were considered indicative of favourable binding.

Cellular thermal shift assay (CETSA) and drug affinity responsive target stability (DARTS) were conducted as previously described [31]. HepG2 cell lysates were treated with or without methyl palmitate and subjected either to graded temperature exposure or to increasing concentrations of pronase E. PPARα protein stability under these conditions was evaluated by Western blot analysis.

#### 2.2.6 RT‐qPCR

We extracted total RNA from treated cells, and assessed its purity and concentration. RNA was reverse transcribed into cDNA, and quantitative PCR was carried out to measure target gene mRNA expression. Relative transcript levels were calculated using the 2^−ΔΔCT^ method, with *Actb* as the internal reference [32]. Primers are listed in the Supporting Informations.

### 2.3 Biochemical Assays

We collected serum, liver tissues, cells, and culture supernatants for biochemical analyses. ALT, AST, TG and TC levels in serum or supernatants were measured using commercial assay kits [33]. ADH and ALDH activities were quantified in liver or cell lysates. Levels of IL‐1β, IL‐6, TNF‐α, SOD, GSH‐Px, MDA, 4‐HNE and ROS in liver tissues were measured according to the manufacturers' protocols.

### 2.4 Western Blot

Liver tissues or cultured cells were lysed, and total protein concentrations were determined using a BCA assay. We separated equal amounts of protein by SDS‐PAGE and transferred them to PVDF membranes. We subsequently blocked membranes with non‐fat milk and incubated them with primary antibodies (overnight, 4°C). Membranes were then incubated with secondary antibodies at room temperature for 2 h. Signals were detected using ECL reagents and imaged. Band intensities were quantified with ImageJ software.

### 2.5 Statistical Analysis

Data were analysed using GraphPad Prism (10.6) and are expressed as mean ± SD. The Shapiro–Wilk test was used to assess normality. Depending on data distribution and experimental design, statistical comparisons were performed using unpaired Student's *t*‐test, one‐way ANOVA, or two‐way ANOVA; Welch's correction was applied when variance heterogeneity was detected. *p* < 0.05 was considered statistically significant.

## 3 Results

### 3.1 Chemical Profiling of YJSB

LC–MS analysis was performed in both positive and negative modes to detect the chemical profile of YJSB. Identification relied on retention time and the protonated molecular ion peak, which were compared with literature and database data. In total, benzaldehyde, isoliquiritin, docosanoic acid, ethyloctadecanoate, methyl palmitate and stearic acid were tentatively identified (Table 1).

**TABLE 1:** Detailed information of YJSB prototype compounds in plasma.

No	Identification	Formula	Adducts	Theoretical *m/z*	Mass Error(ppm)	RT (min)
1	Benzaldehyde	C_7_H_6_O	M—	106.0425	−0.09	11.67
2	Isoliquiritin	C_21_H_22_O_9_	(M‐H)—	417.1192	−0.07	10.05
3	Docosanoic acid	C_22_H_44_O_2_	(M + NH_4_) + [−H_2_O]	340.3575	0.42	22.23
4	Ethyloctadecanoate	C_20_H_40_O_2_	(M + NH_4_) + [−H_2_O]	312.3260	−0.14	20.62
5	Methyl palmitate	C_17_H_34_O_2_	(M + NH_4_) + [−H_2_O]	270.2793	0.53	18.99
6	Stearic acid	C_18_H_36_O_2_	(M + NH_4_) + [−H_2_O]	284.2949	0.2	14.02

### 3.2 YJSB Ameliorates Hepatic Injury in ALD Mice

We established an ALD mouse model using chronic ethanol feeding combined with an acute binge ethanol challenge. Relative to healthy controls, ALD mice showed marked body weight loss, an increased liver‐to‐body weight ratio (liver index), and elevated serum ALT, AST, TG and TC levels, reflecting significant hepatic dysfunction. YJSB administration improved all of these parameters in a dose‐dependent manner (Figure 1A–F). Histopathological evaluation revealed classic ALD‐associated liver lesions, including widespread hepatocyte ballooning, pyknotic nuclei, inflammatory cell infiltration, and substantial lipid accumulation. YJSB treatment markedly attenuated these abnormalities, with the strongest improvement observed in the high‐dose group (Figure 1G–J). In addition, YJSB increased hepatic ADH and ALDH activities (Figure 1K,L), suppressed CYP2E1 expression (Figure 1M,N), elevated SOD and GSH‐Px activities, and reduced hepatic MDA, 4‐HNE and ROS levels (Figure 2A–E). YJSB also significantly decreased pro‐inflammatory cytokines, including IL‐1β, IL‐6 and TNF‐α (Figure 2F–H). These results indicate that YJSB enhances ethanol metabolism while exerting robust antioxidant and anti‐inflammatory effects. Silybin served as a positive control, and high‐dose YJSB produced therapeutic effects comparable to those of silybin. Collectively, these findings demonstrate that YJSB effectively mitigates alcohol‐induced liver injury and represents a promising candidate for ALD therapy.

**FIGURE 1:**
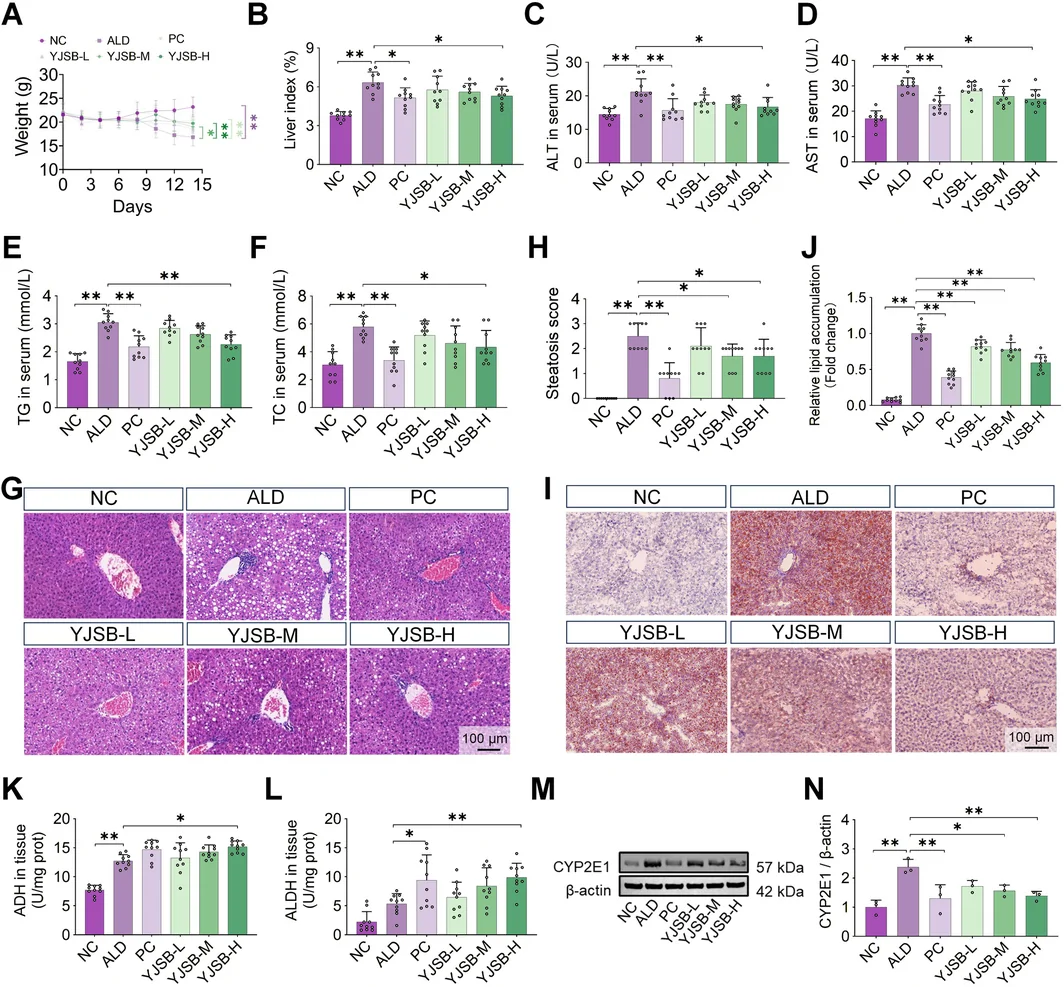
Therapeutic Effects of YJSB on ALD. (A) Starting from the sixth day of modelling, the body weight changes of mice were recorded every 3 days; (B) Liver‐to‐body weight ratio (liver index) determined at sacrifice; (C–F) Serum levels of alanine aminotransferase (ALT), aspartate aminotransferase (AST), triglyceride (TG), and total cholesterol (TC), serving as biochemical indicators of hepatocellular injury and hepatic lipid dysregulation; (G, H) H&E staining and hepatic steatosis scores of liver sections; (I, J) Oil Red O staining and relative positive‐area quantification of liver sections; (K, L) ADH and ALDH activities in liver tissues; (M, N) CYP2E1 protein expressions in liver tissues. Groups: NC, normal control; ALD, ALD model; PC, positive control; YJSB‐L, YJSB low‐dose group; YJSB‐M, YJSB medium‐dose group; YJSB‐H, YJSB high‐dose group. All data are presented as means ± SD, *n* = 10 per group for (A–L), *n* = 3 per group for (M, N), *p < 0.05, **p < 0.01.

**FIGURE 2:**
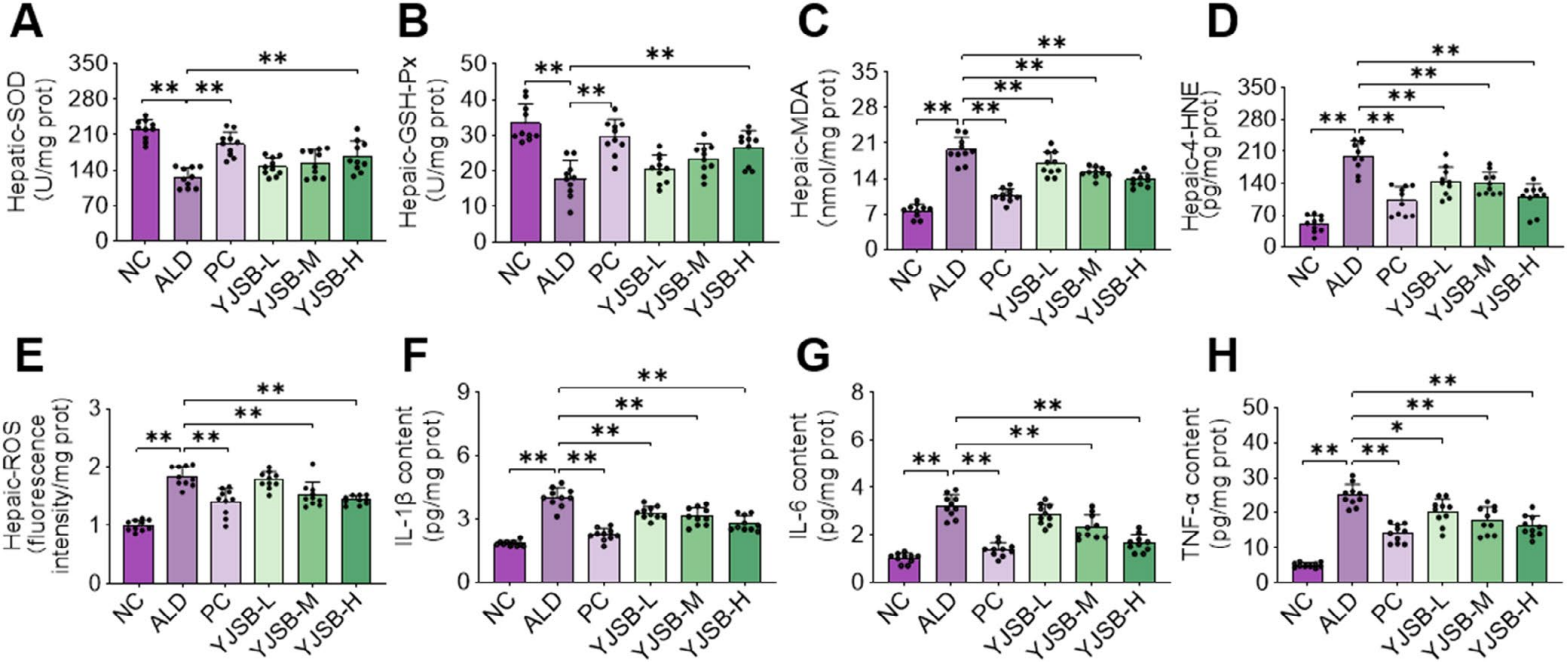
YJSB Inhibits Oxidative Stress and Inflammatory Responses. (A–E) Hepatic oxidative stress markers: (A) SOD activity, (B) GSH‐Px activity, (C) MDA content, (D) 4‐HNE content, and (E) ROS levels. (F–H) Hepatic levels of pro‐inflammatory cytokines: (F) IL‐1β, (G) IL‐6, and (H) TNF‐α measured by ELISA. *n* = 10 per group, *p < 0.05, **p < 0.01.

### 3.3 YJSB Exerts Anti‐ALD Effects by Modulating Arachidonic Acid Metabolism

To elucidate the molecular basis of YJSB's hepatoprotective effects, we performed transcriptomic analysis on liver tissues from the NC, ALD and YJSB‐H groups. DEGs were identified using |log_2_FC| > 1 and *p* < 0.05 (Figure 3A,B). KEGG enrichment analysis of DEGs from both ALD versus NC and YJSB‐H versus ALD comparisons consistently highlighted “Arachidonic acid metabolism” as a significantly enriched pathway (Figure 3C,D). We next conducted untargeted metabolomic analysis on the same liver samples. PCA revealed tight clustering within groups and clear separation between groups, indicating that YJSB substantially restored the metabolic disturbances induced by chronic ethanol exposure (Figure 4A). PLS‐DA models for both ALD versus NC and YJSB‐H versus ALD comparisons yielded *R* ^2^Y > 0.9, and 200‐permutation tests produced *Q* ^2^ < −0.2, confirming strong model reliability without overfitting (Figure 4B–E). Differential metabolites were identified using VIP > 1.0, *p* < 0.05, and FC > 1.5 or < 0.67 (Figure 4F,G). KEGG enrichment of these metabolites was integrated with transcriptomic KEGG enrichment through a Venn diagram, revealing three shared pathways: Arachidonic acid metabolism, Steroid hormone biosynthesis, and Biosynthesis of unsaturated fatty acids (Figure S1). Among these, “Arachidonic acid metabolism” was identified as the most significantly perturbed pathway shared between the two comparisons, strongly implicating this pathway as a central mediator of YJSB's anti‐ALD activity (Figure 4H,I).

**FIGURE 3:**
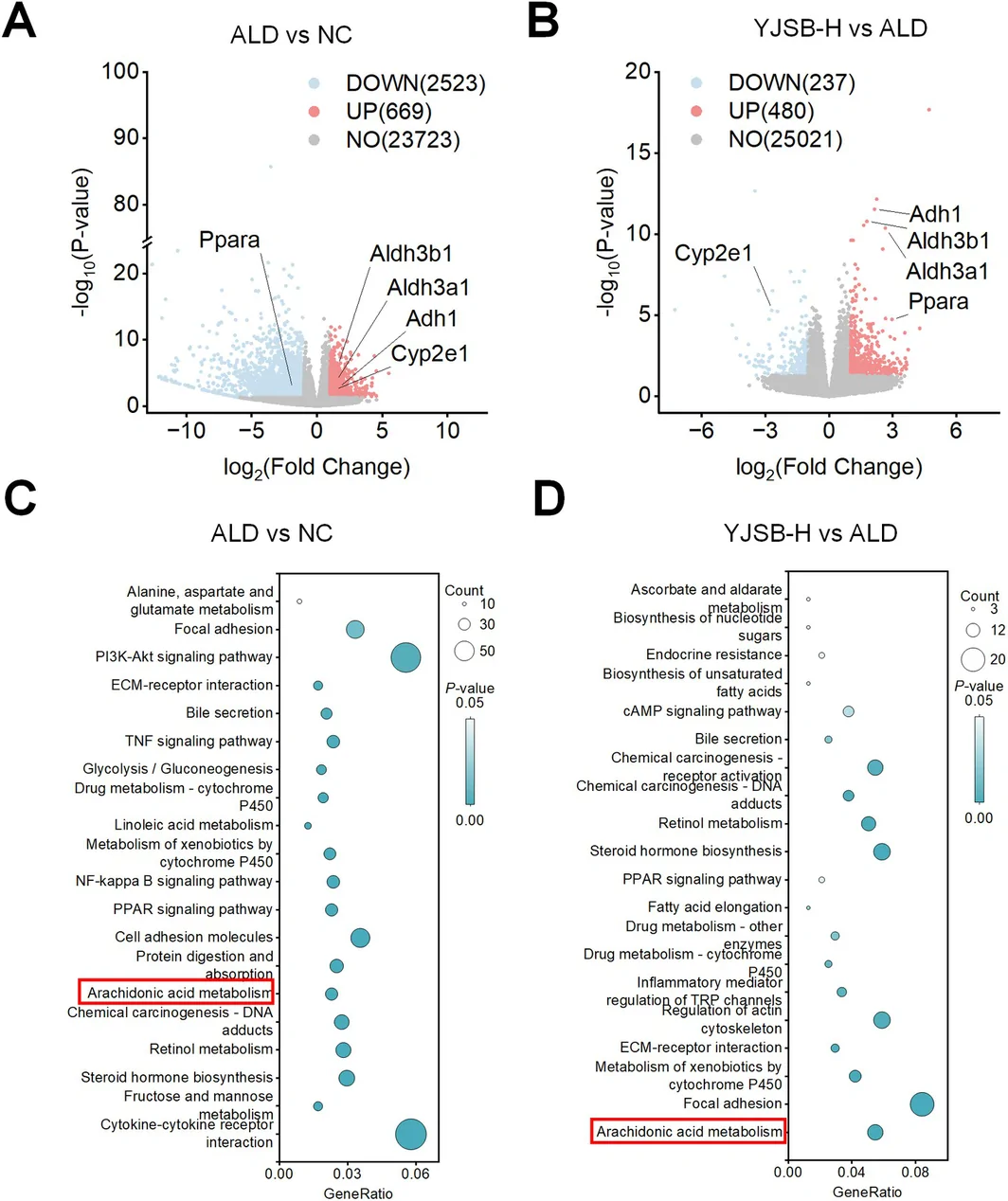
Transcriptomic Effects of YJSB in Livers of ALD Mice. (A, B) Differentially expressed genes (DEGs) identified by RNA sequencing analysis (screening criteria: |log_2_ FC| > 1 and *p* < 0.05) in the comparisons of ALD versus NC (A) and YJSB‐H versus ALD (B). (C, D) KEGG enrichment analysis of DEGs from the ALD versus NC comparison (C) and the YJSB‐H versus ALD comparison (D), displaying the top significantly enriched pathways. “Arachidonic acid metabolism” pathway (highlighted) was identified as significantly enriched in both comparisons. *n* = 6 per group.

**FIGURE 4:**
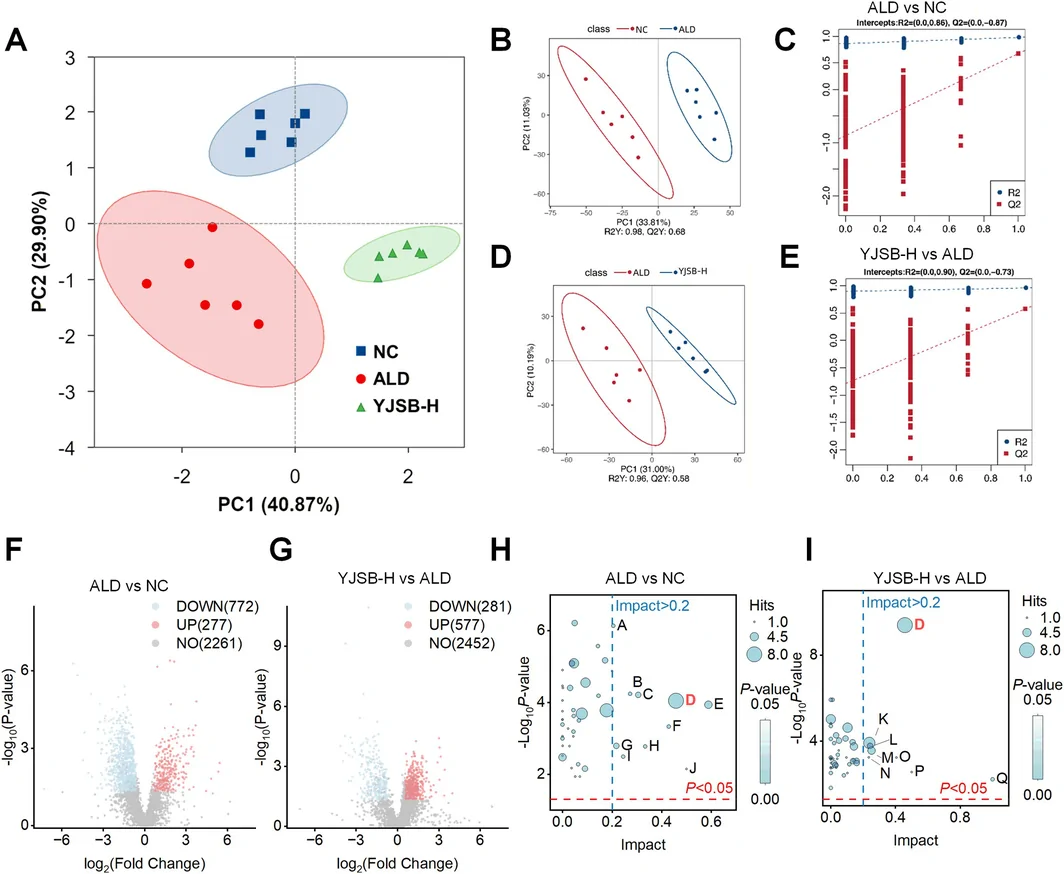
Untargeted Metabolomic Effects of YJSB in Livers of ALD Mice. (A) PCA analysis of metabolites from NC, ALD, and YJSB‐H groups; (B–E) PLS‐DA scores and 200 permutation tests for ALD versus NC and YJSB‐H versus ALD; (F, G) Identification of differentially abundant metabolites (screening criteria: Variable importance in projection VIP > 1.0, *p* < 0.05, and FC > 1.5 or < 0.67) in ALD versus NC (F) and YJSB‐H versus ALD (G) comparisons. (H, I) KEGG enrichment analysis of differential metabolites from ALD versus NC and YJSB‐H versus ALD comparisons. “Arachidonic acid metabolism” (highlighted) emerged as the most significantly enriched pathway. Key enriched pathways are labelled as follows: A, Pentose and glucuronate interconversions; B, Ubiquinone and other terpenoid‐quinone biosynthesis; C, Alanine, aspartate and glutamate metabolism; D, Arachidonic acid metabolism; E, Vitamin B6 metabolism; F, Taurine and hypotaurine metabolism; G, Glycerophospholipid metabolism; H, alpha‐Linolenic acid metabolism; I, Arginine biosynthesis; J, Riboflavin metabolism; K, Citrate cycle (TCA cycle); L, Pentose phosphate pathway; M, Glyoxylate and dicarboxylate metabolism; N, Nicotinate and nicotinamide metabolism; O, Ascorbate and aldarate metabolism; P, Phenylalanine, tyrosine and tryptophan biosynthesis; Q, Linoleic acid metabolism. *n* = 6 per group.

Transcriptomic analysis of arachidonic acid metabolism–related genes showed that YJSB markedly downregulated several pro‐inflammatory and pro‐oxidant genes, including *Alox12*, *Alox5*, *Cbr1*, *Cbr3*, *Cyp2b10*, *Cyp2c29*, *Cyp2e1*, *Ephx2* and *Pla2g4a*, all of which contribute to ALD progression [34, 35, 36, 37, 38, 39, 40, 41, 42]. In contrast, YJSB upregulated protective genes such as *Cyp4a14*, *Cbr2*, *Cyp4a12a* and *Ptgis*, which are associated with anti‐inflammatory, antioxidant, and antifibrotic functions [40, 43, 44, 45]. Among these, *Cyp4a14* displayed the most robust increase following YJSB treatment (Figure 5A).

**FIGURE 5:**
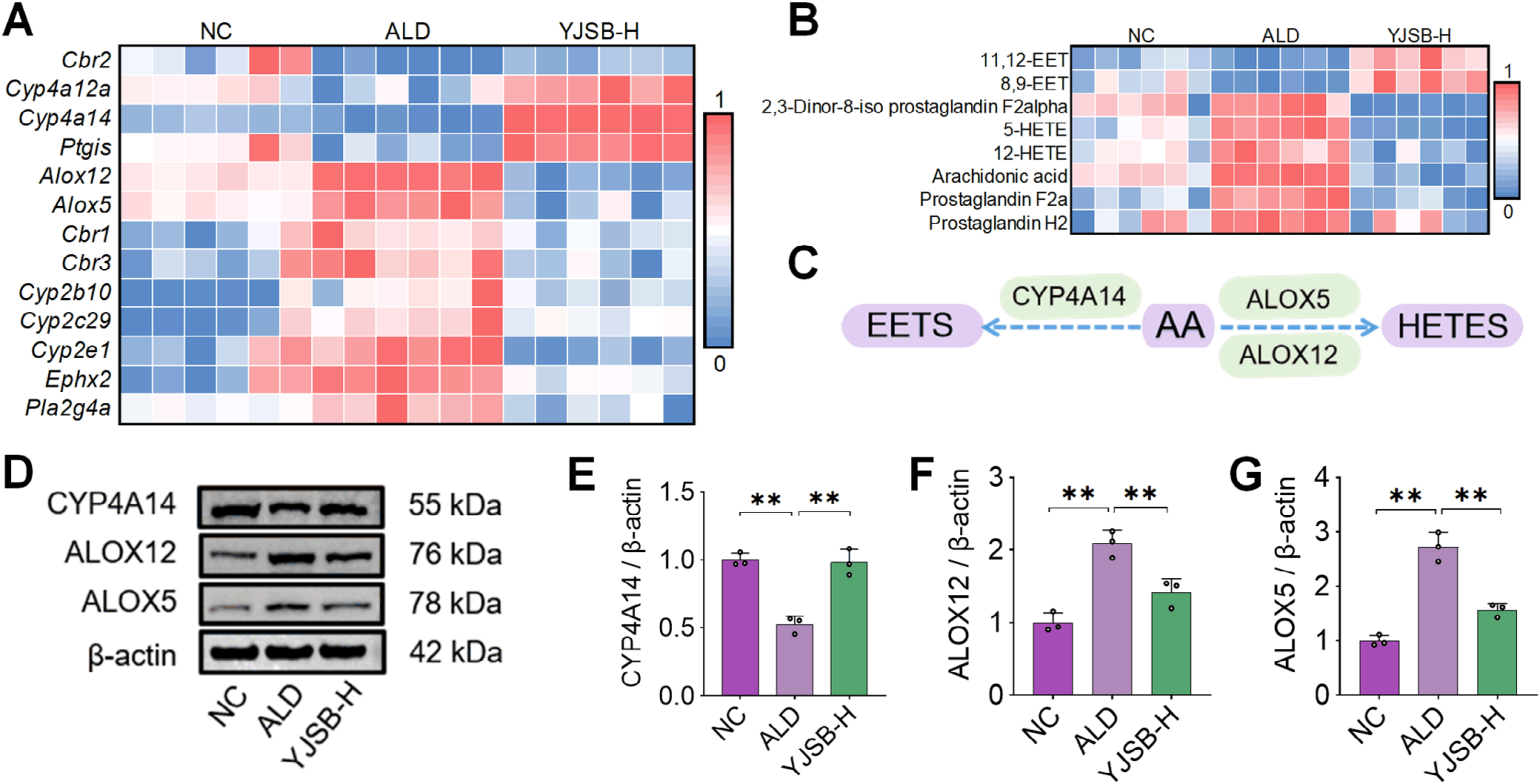
YJSB Regulates Arachidonic Acid Metabolism in Livers of ALD Mice. (A) Heatmap of expression of arachidonic acid metabolism‐related genes from transcriptomics; (B) Heatmap of expression of arachidonic acid metabolism‐related metabolites from untargeted metabolomics; (C) Schematic diagram of arachidonic acid metabolism; (D–G) Western blot analysis (D) and densitometric quantification of CYP4A14 (E), ALOX12 (F), and ALOX5 (G) protein expression in mouse liver tissues. YJSB significantly increased CYP4A14 expression while markedly suppressing ALOX12 and ALOX5 protein levels. *n* = 6 per group for (A, B); *n* = 3 per group for (D–G). **p < 0.01.

Metabolomic profiling of arachidonic acid pathway intermediates further demonstrated that YJSB reduced levels of several pro‐inflammatory and pro‐oxidant metabolites including arachidonic acid [39, 46, 47], 5‐HETE, 12‐HETE, 2,3‐dinor‐8‐iso‐prostaglandin F2α, prostaglandin F2α, and prostaglandin H2 while increasing anti‐inflammatory and antioxidant epoxyeicosatrienoic acids (11,12‐EET and 8,9‐EET) [48, 49] (Figure 5B).

By integrating transcriptomic and metabolomic evidence, we propose that YJSB mitigates ALD by modulating arachidonic acid metabolism, with CYP4A14 acting as a key regulatory enzyme (Figure 5C). In vivo analyses confirmed that YJSB significantly increased CYP4A14 protein expression and suppressed ALOX12 and ALOX5, supporting this mechanistic hypothesis (Figure 5D–G).

### 3.4 Methyl Palmitate Component Alleviates Alcohol‐Induced Hepatocyte Injury In Vitro

To further investigate YJSB's molecular mechanism in vitro, we first characterized its chemical composition and its serum profile using UPLC‐Q‐TOF‐MS. By analysing the total ion chromatograms acquired in both positive‐ and negative‐ion modes and comparing them with literature data, we identified benzaldehyde, isoliquiritin, docosanoic acid, ethyloctadecanoate, methyl palmitate and stearic acid as the active constituents of YJSB and selected them for subsequent in vitro validation (Figure 6A,B).

**FIGURE 6:**
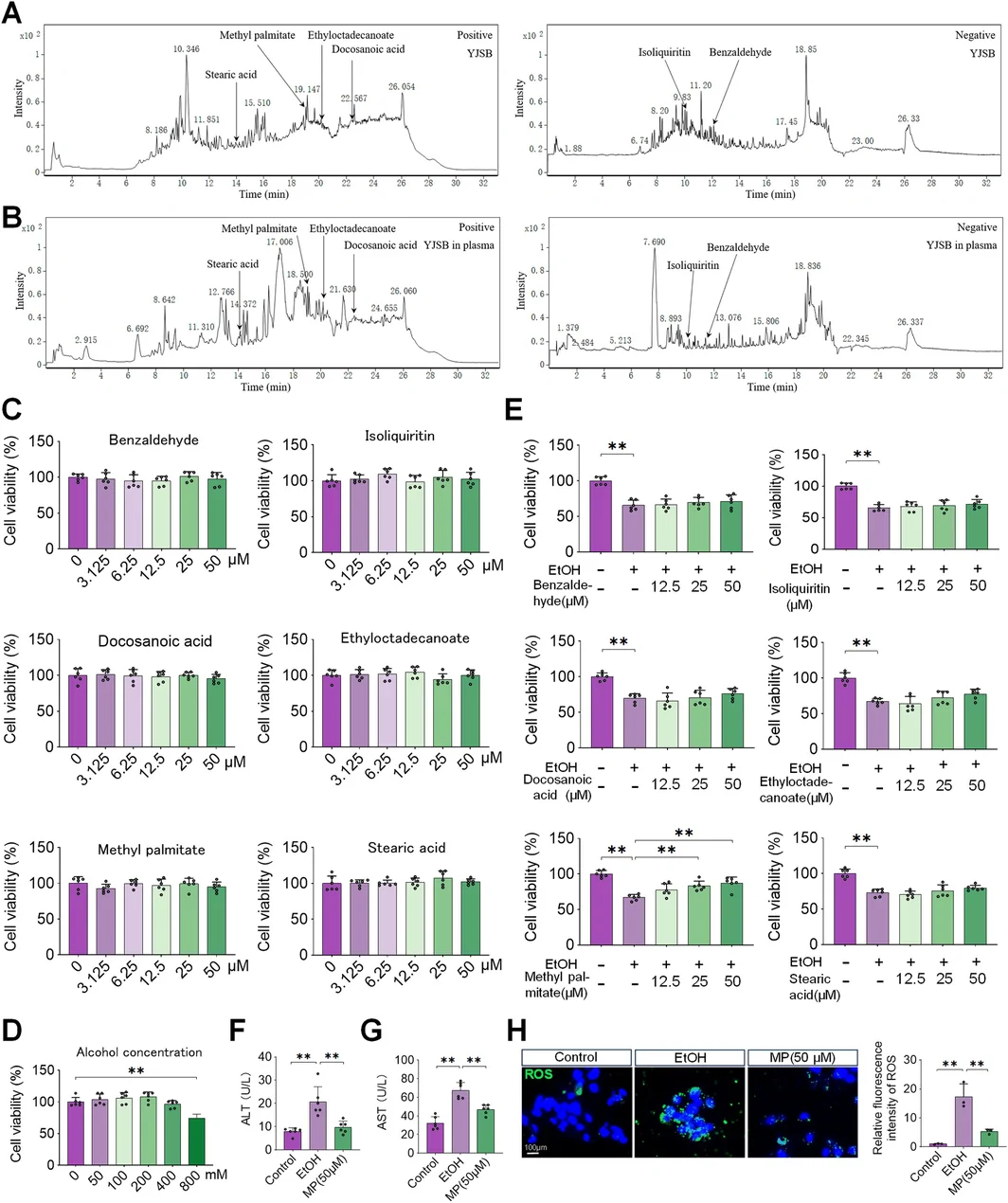
Methyl Palmitate in YJSB Suppresses Oxidative Stress and Improves Alcohol‐Induced Hepatocyte Injury. (A) Total ion chromatogram (TIC) of YJSB extract acquired in positive and negative ion modes; (B) TIC of plasma collected from YJSB‐treated mice in positive and negative ion modes. Six compounds were identified by comparison with reference standards and literature data: Benzaldehyde, isoliquiritin, docosanoic acid, ethyloctadecanoate, methyl palmitate, and stearic acid; (C) The non‐toxic concentrations of six compounds in HepG2 cells were screened by the MTT assay. (D) MTT screening for alcohol concentrations (50–800 mM) inducing hepatocyte injury; (E) Methyl palmitate, ethyloctadecanoate, and stearic acid increase cell viability in alcohol‐treated HepG2 cells, with methyl palmitate showing the most pronounced effect; (F, G) Methyl palmitate decreases ALT (F) and AST (G) activities in supernatants of alcohol‐treated HepG2 cells; (H) Methyl palmitate reduces intracellular ROS fluorescence intensity in alcohol‐treated HepG2 cells. MP, methyl palmitate. *n* = 6 per group for (C–G); *n* = 3 per group for (H). **p < 0.01.

Using HepG2 cells, we determined non‐toxic working concentrations of these candidates with an MTT assay; the safe concentration for benzaldehyde was 50 μM, isoliquiritin was 50 μM, docosanoic acid was 50 μM, ethyloctadecanoate was 50 μM, methyl palmitate was 50 μM, and stearic acid was 50 μM (Figure 6C). We next established an alcohol‐induced hepatocyte injury model by exposing cells to increasing ethanol concentrations and selected 800 mM as the optimal dose to induce ALD‐like damage (Figure 6D). Cells were then treated with graded concentrations of 12.5, 25 and 50 μM. MTT assay results showed that all tested components improved cell viability to varying extents, with methyl palmitate producing the most prominent protective effect (Figure 6E). Accordingly, methyl palmitate was selected as the primary active constituent for mechanistic studies.

Initial experiments demonstrated that methyl palmitate markedly reduced ALT and AST activities in the culture supernatant and lowered intracellular ROS levels (Figure 6F–H), confirming its hepatoprotective activity in vitro.

### 3.5 Methyl Palmitate Activates PPARα to Upregulate CYP4A14 and Exert Anti‐ALD Effects

Based on the in vivo findings, we proposed that methyl palmitate mediates its hepatoprotective effects through CYP4A14 regulation. Western blot analysis confirmed that methyl palmitate increased CYP4A14 protein expression in alcohol‐injured hepatocytes (Figure 7A,B). Because CYP4A14 is a key arachidonic acid–metabolizing enzyme under transcriptional control of PPARα [43], we next examined PPARα involvement.

**FIGURE 7:**
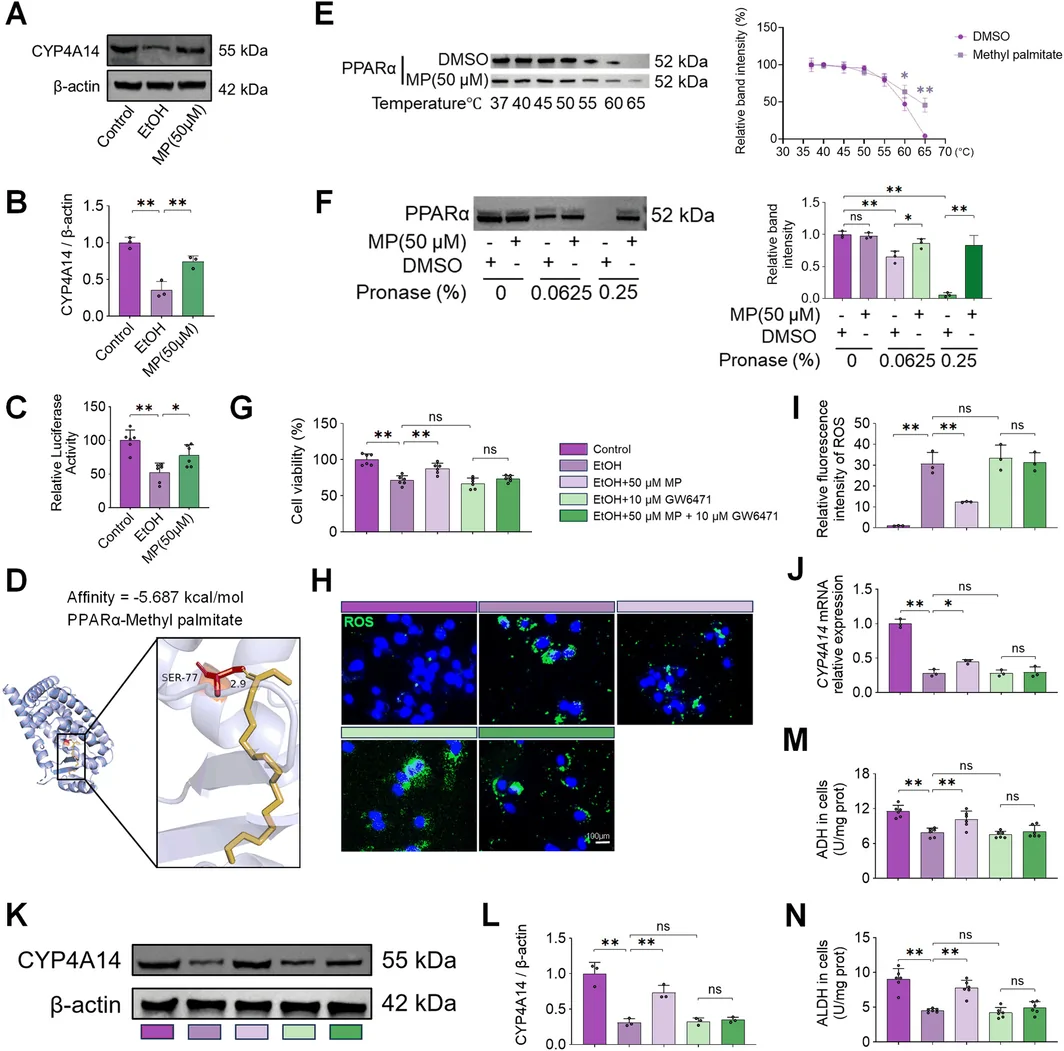
Methyl Palmitate Activates PPARα, Upregulates CYP4A14, and Improves Alcohol‐Induced Hepatocyte Injury. (A, B) Western blot analysis (A) and densitometric quantification (B) demonstrating that methyl palmitate upregulates CYP4A14 protein expression in alcohol‐injured hepatocytes; (C) PPARα transcriptional activity measured by a dual‐luciferase reporter assay indicating that methyl palmitate enhances PPARα transcriptional activity in alcohol‐injured hepatocytes; (D) Molecular docking model showing the predicted binding affinities (−5.687 kcal/mol) between methyl palmitate and PPARα; (E, F) Cellular thermal shift assay (CETSA) and drug affinity responsive target stability (DARTS) assay showing that methyl palmitate stabilizes PPARα against thermal denaturation (E) and proteolytic degradation (F); (G–N) Co‐treatment with PPARα antagonist GW6471 (10 μM) completely abolished the protective effects of MP in ethanol‐injured hepatocytes, including (G) reversal of MP‐mediated improvement in cell viability, (H, I) restoration of intracellular ROS accumulation, (J–L) suppression of CYP4A14 mRNA and protein upregulation, (M, N) blockade of ADH and ALDH activity enhancement in cells. MP, methyl palmitate. *n* = 3 per group for (A, B, E, F, H, I, K, L); *n* = 6 per group for (C, G, J, M, N). *p < 0.05; **p < 0.01; ns, no significance.

Our transcriptomic analysis also identified enrichment of the “PPAR signaling pathway”, with *Ppara* expression significantly elevated following YJSB treatment. Previous studies show that PPARα agonists enhance hepatic ADH1 expression and activity [50], and PPARα ligands induce ALDH expression—both essential for efficient ethanol metabolism [51].

We therefore assessed whether methyl palmitate activates PPARα. A luciferase reporter assay demonstrated that methyl palmitate significantly increased PPARα transcriptional activity in ethanol‐injured hepatocytes (Figure 7C). Molecular docking using CB‐DOCK2 predicted strong binding between methyl palmitate and PPARα (affinity = −5.687 kcal/mol) (Figure 7D). CETSA and DARTS assays further supported this interaction, showing increased thermal and proteolytic stability of PPARα in the presence of methyl palmitate (Figure 7E,F).

To test functional relevance, we co‐treated ethanol‐injured hepatocytes with methyl palmitate and the PPARα antagonist GW6471 (10 μM). GW6471 eliminated the protective effects of methyl palmitate, reversing improvements in cell viability, restoring ROS accumulation, suppressing CYP4A14 induction, and blocking the enhancement of ADH and ALDH activities (Figure 7G–N).

Together, these data show that methyl palmitate, a bioactive component of YJSB, acts through PPARα activation to upregulate CYP4A14 and modulate arachidonic acid metabolism, thereby reducing oxidative stress during alcohol‐induced hepatocyte injury.

## 4 Discussion

The incidence of ALD continues to rise with rapid lifestyle changes and widespread alcohol consumption. ALD encompasses a spectrum ranging from hepatic steatosis and hepatitis to cirrhosis and imposes a substantial healthcare burden [52]. Beyond liver injury, patients frequently experience fatigue, anorexia, and abdominal distension, symptoms that impair daily functioning and social engagement. These clinical manifestations often trigger anxiety, depression, and other psychological disorders, further diminishing quality of life [53]. Despite the growing prevalence of ALD, effective pharmacotherapies remain limited, highlighting the need for new, reliable, and safe therapeutic options. The NIAAA/Gao‐binge model is one of the most widely used and reliable experimental systems for studying ALD [25]. This model combines chronic ethanol feeding with a final acute ethanol challenge, closely resembling human patterns of excessive drinking and capturing the pathophysiology of acute‐on‐chronic liver injury. It can induce hepatic steatosis, inflammation, and even early features of hepatitis within a short experimental period.

In our study, model mice displayed hallmark manifestations of ALD, including hepatic dysfunction, pronounced histopathological injury, elevated inflammatory and oxidative stress markers, and disrupted expression of key alcohol‐metabolizing enzymes, confirming successful model establishment. YJSB treatment significantly improved these pathological features. It alleviated liver dysfunction, reduced hepatic inflammation and oxidative stress, restored ethanol‐metabolizing enzyme activity, and decreased CYP2E1 expression–a major ethanol‐inducible, ROS‐generating enzyme [54, 55]. Notably, high‐dose YJSB exhibited therapeutic efficacy comparable to silybin, a well‐validated clinical hepatoprotective agent, underscoring YJSB's potential as an effective anti‐ALD therapy. Furthermore, the in vitro activity of methyl palmitate–the principal bioactive constituent of YJSB–provides mechanistic support for its hepatoprotective effects.

Integrated transcriptomic and untargeted metabolomic analyses consistently identified arachidonic acid (AA) metabolism as the central pathway mediating the hepatoprotective effects of YJSB. AA, an ω‐6 polyunsaturated fatty acid stored in membrane phospholipids, is rapidly released under ethanol‐induced oxidative stress. Once liberated, AA is metabolized through the cyclooxygenase (COX), lipoxygenase (LOX), and cytochrome P450 (CYP) pathways, generating ROS and pro‐inflammatory mediators. These metabolites amplify oxidative stress and inflammation, thereby accelerating alcohol‐induced liver injury [56, 57, 58]. Previous reports confirm that modulating AA metabolism can mitigate liver damage [59, 60]. In line with these findings, our data showed that key AA‐metabolizing enzymes such as ALOX12 and ALOX5–potent drivers of oxidative and inflammatory injury–were markedly upregulated in ALD mouse livers, whereas YJSB treatment effectively reversed this pathological activation.

Among AA‐related enzymes, CYP4A14 emerged as particularly significant. YJSB strongly upregulated hepatic CYP4A14 expression and increased downstream anti‐inflammatory epoxyeicosatrienoic acids (EETs). CYP4A14, a major CYP4A isoform expressed in murine liver, catalyses the ω‐hydroxylation of medium‐chain fatty acids and AA [61]. A previous study demonstrated that CYP4A14 activation reduces hepatic steatosis, inflammation, and metabolic dysregulation, whereas CYP4A14 loss‐of‐function abolishes these benefits [43]. Other reports show that CYP4A14 upregulation exerts antifibrotic effects in cholestatic liver disease [62]. However, context‐dependent effects have also been documented: in methionine‐choline‐deficient (MCD) diet–induced fibrosis, CYP4A14 activation aggravated disease progression, while *Cyp4a14* knockout conferred protection [63]. These conflicting observations underscore the dual, condition‐specific nature of CYP4A14 in liver pathology and highlight the need for precise therapeutic targeting. Our findings demonstrate a protective role for CYP4A14 in ALD and provide mechanistic evidence that YJSB confers hepatoprotection, at least in part, by modulating CYP4A14 within the AA metabolic network.

CYP4A14 is a well‐recognized transcriptional target of PPARα [43]. Genetic deletion of PPARα markedly reduces hepatic Cyp4a gene expression in mice, particularly *Cyp4a14* [64]. As a ligand‐activated nuclear receptor, PPARα is highly expressed in the liver and regulates oxidative stress, inflammation, lipid homeostasis, and fibrogenesis [65]. Chronic ethanol exposure suppresses PPARα activity, leading to heightened oxidative stress and exacerbation of ALD [66]. The protective effects of PPARα activation are well documented in metabolic dysfunction‐associated steatotic liver disease (MASLD) [43, 67], and emerging evidence supports its role in reducing inflammation and fibrosis during ALD [68]. In addition, PPARα agonists increase hepatic ADH1 levels and activity [50], while PPARα ligands induce ALDH expression—both essential for efficient ethanol metabolism [51]. These findings align with our data showing that YJSB and its active component methyl palmitate activate PPARα. Notably, the PPARα antagonist GW6471 eliminated the protective effects of methyl palmitate in vitro, confirming that PPARα activation is a central mechanism through which YJSB mitigates ALD.

In summary, this study demonstrates that methyl palmitate, the principal bioactive constituent of the Dai medicinal formulation YJSB, directly activates PPARα, upregulates its downstream target CYP4A14, reprograms hepatic arachidonic acid metabolism, reduces oxidative stress, and consequently mitigates ALD‐related liver injury. Despite these advances, the precise chemical determinants within YJSB that shift the context‐dependent, “double‐edged” behaviour of CYP4A14 toward a protective phenotype remain unresolved. Future research should integrate multi‐omics strategies and multi‐component, multi‐target analyses to clarify these mechanisms. Moreover, although methyl palmitate's activation of the PPARα–CYP4A14 axis highlights its therapeutic promise, comprehensive evaluation of its in vivo dose–response–toxicity characteristics and its potential interactions with current ALD treatments is essential to support precision application in clinical settings.

## 5 Conclusion

This work confirms that YJSB confers significant protection against ALD. At the mechanistic level, the active component methyl palmitate directly engages and activates PPARα, leading to CYP4A14 upregulation, modulation of arachidonic acid metabolism, attenuation of oxidative stress, and alleviation of alcohol‐induced liver injury (Figure 8). These findings provide the first clear delineation of the “component–target” mechanism underlying YJSB's anti‐ALD activity and offer a novel therapeutic strategy, as well as a material basis, for the broader application of traditional ethnic medicines in ALD treatment.

**FIGURE 8:**
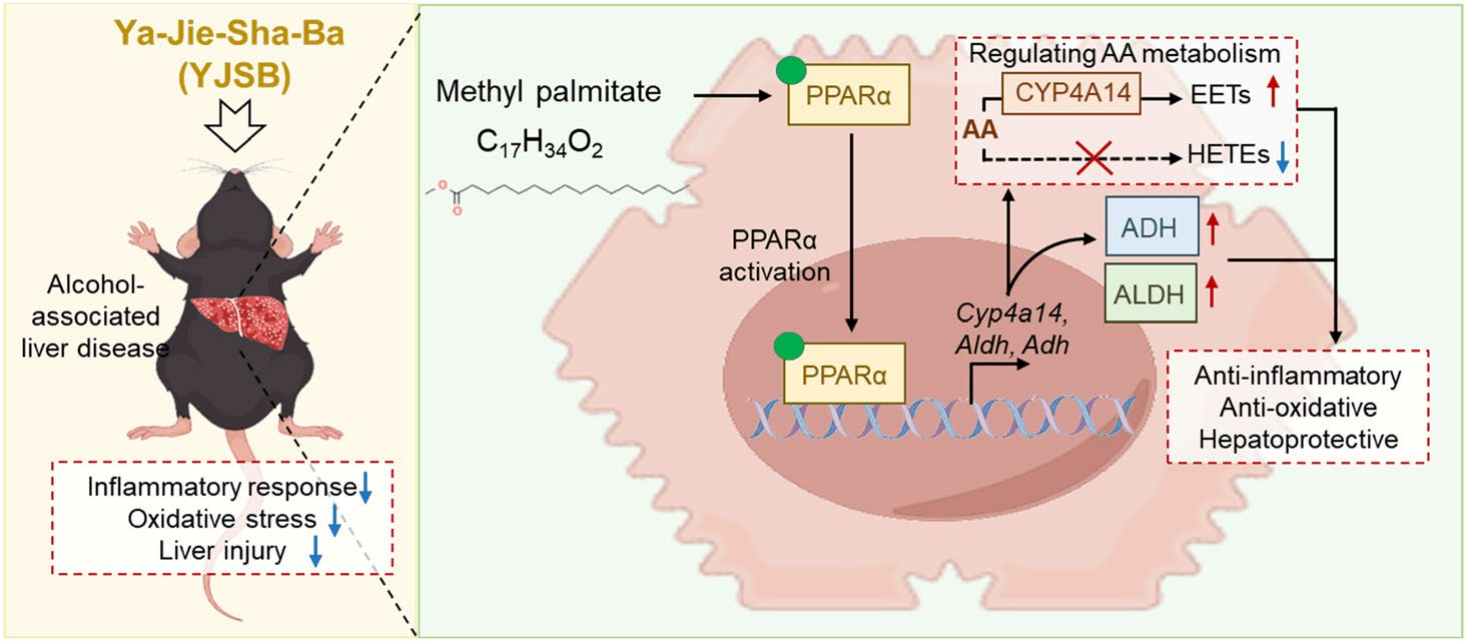
Schematic Representation of the Anti‐ALD Mechanisms of YJSB. Methyl palmitate in YJSB activates PPARα, upregulates CYP4A14 expression, regulates arachidonic acid metabolism, reduces oxidative stress, and alleviates ALD‐induced liver injury.

## Author Contributions

**Dehong Ma:** writing – original draft, funding acquisition. **Xia Chen:** writing – original draft. **Huixian Dao:** writing – original draft, data curation, formal analysis. **Hongyu Deng:** data curation, formal analysis, investigation. **Yingcheng Guo:** investigation, data curation. **Hui Yang:** software, investigation. **Yuanyuan Chen:** methodology. **Congmei Zhang:** formal analysis, methodology. **Yuan Long:** formal analysis, data curation. **Weibo Wen:** project administration, writing – review and editing. **Yinghong Zhao:** conceptualization, writing – review and editing. **Huantian Cui:** project administration, writing – review and editing.

## Funding

This work was supported by Project for Cultivating Qihuang Scholars, Leading Talents in Traditional Chinese Medicine, 2025‐182. Xingdian Talents Support Program‐Expert Physicians Program, XDYC‐MY‐2022‐0022. Xingdian Talents Support Program‐Youth Talent Program, XDYC‐QNRC‐2024‐518. Caiyun Postdoctoral Program—Non‐employed Postdoctoral Researcher Funding Project/Young Talent Cultivation Program of China Association of Chinese Medicine, 2025‐QNRC2‐B46. Open Research Fund Program of Yunnan Key Laboratory for Dai and Yi Medicines (Yunnan University of Chinese Medicine), 2025ZD2502, 2025SS2514.

## Ethics Statement

All animals were housed in the Animal Experiment Center of Yunnan University of Chinese Medicine and acclimated for one week before experimentation. All procedures complied with national guidelines for the ethical use of laboratory animals and were approved by the Yunnan University of Chinese Medicine Ethics Committee (Approval No.: YNUCM‐XMSB‐G‐20250274).

## Consent

All authors agree to publish this article.

## Conflicts of Interest

The authors declare no conflicts of interest.

## Supporting information


**Figure S1:** Integrated transcriptomics and metabolomics analysis identifies convergent KEGG enrichment pathways modulated by YJSB in ALD. Venn diagram showing the overlap of significantly enriched KEGG pathways from transcriptomics and metabolomics analyses. The central intersection highlights three shared pathways: Arachidonic acid metabolism, Steroid hormone biosynthesis, and Biosynthesis of unsaturated fatty acids.

## Data Availability

The data that support the findings of this study are available from the corresponding author upon reasonable request.
