# Supplementary material for: Ya‐Jie‐Sha‐Ba Decoction Regulates Arachidonic Acid Metabolism to Treat Alcohol‐Associated Liver Disease by Activating PPARα–CYP4A14 Axis: Insights From Multi‐Omics Analysis and Experimental Validations
Source: J Cell Mol Med. 2026 Sep 29;30(19):e71385. doi: 10.1111/jcmm.71385 (PMC13624284; doi:10.1111/jcmm.71385)
Supplement: Supplementary file 1 [file JCMM-30-e71385-s001.docx]

**Supplementary Materials**

**1 Reagents**

SOD (A001-3-2), MDA (A003-1-2), GSH-Px (A006-2-1), ALT (C009-2-1), AST (C010-2-1), TG (A110-1-1), TC (A111-1-1) and Total protein (A045-4-2) were obtained from Nanjing Jiancheng Biological Engineering Institute. ADH (S0241S) and ALDH (S0568S) were obtained from Beyotime Co., Ltd.. 4-HNE (ab287803) was obtained from Abcam (Cambridge, UK). Elisa kit for IL-6 (ml063159), IL-1β (ml063132) and TNF-α (ml002095) were purchased from Shanghai Enzyme-linked Biotechnology Co., Ltd. CYP2E1 (19937-1-AP), ALOX5 (10021-1-Ig) and PPARA (66826-1-Ig) were obtained from proteintech Co., Ltd.. CYP4A14 (sc-46087) was purchased from santacruze Co., Ltd.. ALOX12(E3O9P) was purchased from Cell Signaling Technology Co., Ltd..

**2 Qualitative analysis of Ya-Jie-Sha-Ba**

**2.1 Instruments**

Agilent 1290 Infinity II Ultra-High Performance Liquid Chromatography (UHPLC) system (Agilent, USA); high-resolution mass spectrometer (Q-TOF G6545, Agilent, USA); chromatographic column: Agilent EclipsePlus C18 RRHD (1.8 μm, 2.1 mm × 100 mm).

**2.2 Chromatographic Conditions**

Chromatographic separation was performed on an Agilent 1290 Infinity II UHPLC system equipped with an Agilent EclipsePlus C18 column (2.1 mm × 100 mm, 1.8 μm).

In positive ion mode, the mobile phase consisted of (A) 0.1% formic acid in water and (B) 0.1% formic acid in acetonitrile, using a gradient elution.

In negative ion mode, the mobile phase consisted of (A) 0.01% formic acid in water and (B) 0.01% formic acid in acetonitrile, also under gradient elution.

The gradient program was as follows:

0–5.0 min, 2% B;

5.0–20.0 min, 2% → 100% B;

20.0–25.0 min, 100% B;

25.0–30.0 min, 100% → 2% B;

30.0–33.0 min, 2% B.

Column temperature: 40 °C; flow rate: 0.4 mL/min; injection volume: 5 μL.

**2.3 Mass Spectrometric Conditions**

An electrospray ionization (ESI) source was used for data acquisition in both positive and negative ion modes. Drying gas temperature: 320 °C; drying gas flow: 8 L·min⁻¹; Sheath gas temperature: 350 °C; sheath gas flow: 11 L·min⁻¹; Nebulizer pressure: 35 psi; Nozzle voltage: 3.5 kV; Capillary voltage: 1000 V; Mass scan range: *m/z* 100–1000; Collision energy: 10, 20, and 40 eV (stepped); High-purity nitrogen was used as the nebulizer gas, drying gas, and sheath gas.

**2.4 Data analysis**

The MS data was processed by Agilent MassHunter Workstation Qualitative Analysis 10.0 (Agilent, USA) with the steps of raw data introduction, peak extraction and adduct deconvolution. The identification was finally determined by in consideration of retention time error of reference substance, mass error of mother ion, match degree of daughter ions, isotope distribution and peak area after searching the reference substance database (Agilent- Natural Standard NPs PCDL, NatureStandard Shanghai Standard Technology Co., Ltd.) and theoretical database constructed by literature and public databases.

**3 Supplementary figures**

**Figure S1. Integrated transcriptomics and metabolomics analysis identifies convergent KEGG enrichment pathways modulated by YJSB in ALD.** Venn diagram showing the overlap of significantly enriched KEGG pathways from transcriptomics and metabolomics analyses. The central intersection highlights three shared pathways: Arachidonic acid metabolism, Steroid hormone biosynthesis, and Biosynthesis of unsaturated fatty acids.
